# Diet, weight management, physical activity and Ovarian & Breast Cancer Risk in women with *BRCA1/2* pathogenic Germline gene variants: systematic review

**DOI:** 10.1186/s13053-020-0137-1

**Published:** 2020-03-07

**Authors:** Adriana M. Coletta, Susan K. Peterson, Leticia A. Gatus, Kate J. Krause, Susan M. Schembre, Susan C. Gilchrist, Banu Arun, Y. Nancy You, Miguel A. Rodriguez-Bigas, Larkin L. Strong, Karen H. Lu, Karen Basen-Engquist

**Affiliations:** 1 Department of Behavioral Science, The University of Texas MD Anderson Cancer Center, P.O. Box 301439, Unit 1330, Houston, TX 77030-1439 USA; 2 Cancer Control and Population Sciences Program, Huntsman Cancer Institute, Salt Lake City, UT USA; 3 Department of Health, Kinesiology, and Recreation, The University of Utah, Salt Lake City, UT USA; 4 Research Medical Library, The University of Texas MD Anderson Cancer Center, Houston, TX 77030 USA; 5 Department of Family and Community Medicine, College of Medicine- Tucson, University of Arizona, Tucson, AZ 85724 USA; 6 Department of Clinical Cancer Prevention, The University of Texas MD Anderson Cancer Center, Houston, TX 77030 USA; 7 Department of Breast Medical Oncology, The University of Texas MD Anderson Cancer Center, Houston, TX 77030 USA; 8 Department of Surgical Oncology, The University of Texas MD Anderson Cancer Center, Houston, TX 77030 USA; 9 Department of Health Disparities Research, The University of Texas MD Anderson Cancer Center, Houston, TX 77030 USA; 10 Department of Gynecologic Oncology and Reproductive Medicine, The University of Texas MD Anderson Cancer Center, Houston, TX 77030 USA

**Keywords:** BRCA, Breast Cancer, Ovarian Cancer, Diet, Physical activity, Weight

## Abstract

**Introduction:**

Women with pathogenic germline gene variants in *BRCA1* and/or *BRCA2* are at increased risk of developing ovarian and breast cancer. While surgical and pharmacological approaches are effective for risk-reduction, it is unknown whether lifestyle approaches such as healthful dietary habits, weight management, and physical activity may also contribute to risk-reduction. We conducted a systematic review of evidence related to dietary habits, weight status/change, and physical activity on ovarian and breast cancer risk among women with *BRCA1/2* pathogenic variants.

**Methods:**

We searched Medline, EMBASE, CENTRAL, PubMed, and clinicaltrials.gov up to October 3, 2019. We identified 2775 records and included 21.

**Results:**

There is limited evidence related to these factors and ovarian cancer risk. For breast cancer risk, evidence suggests higher diet quality, adulthood weight-loss of ≥10 pounds, and activity during adolescence and young-adulthood may be linked with decreased risk. Higher meat intake and higher daily energy intake may be linked with increased risk.

**Conclusions:**

There is not enough evidence to suggest tailored recommendations for dietary habits or weight management among women with *BRCA1/2* pathogenic variants compared to the general population for ovarian and breast cancer risk-reduction, and physical activity recommendations should remain the same.

## Background

The estimated risk up to 80 years of age for ovarian and breast cancer among women with *BRCA1* and *BRCA2* pathogenic germline gene variants is 44 and 72% respectively for *BRCA1* and 17 and 69% respectively for *BRCA2* [1]. Effective surgical and pharmacological approaches are available for risk-reduction, such as risk-reducing surgery in the context of both breast and ovarian cancer, chemoprevention in the context of breast cancer, and oral contraceptive use in the context of ovarian cancer [2, 3]. What is currently unknown is whether complementary lifestyle approaches such as dietary habits, weight management, and physical activity (PA) may also contribute to cancer risk-reduction among this group of high-risk women.

To date, there are five reviews evaluating the impact of either dietary habits, weight management, or PA, or a combination of only two of these factors (i.e. diet and weight), on ovarian and/or breast cancer risk among women with *BRCA1/2* pathogenic germline gene variants from 1997 to 2015 [3–7]. Four of the five previous reviews included women with *BRCA1* and *BRCA2* pathogenic germline gene variants, but only in the context of breast cancer risk [3, 5–7]. And only one review assessed ovarian cancer risk, and this was only in relation to alcohol intake [6]. No studies have exclusively evaluated healthful dietary habits, weight management, and PA together as they relate to both ovarian and breast cancer risk in this high-risk population.

The purpose of the current systematic review was to explore the state of evidence related to these lifestyle factors and ovarian and breast cancer risk among women with *BRCA1/2* pathogenic germline gene variants, in order to determine the extent to which lifestyle recommendations should differ compared to the general population.

## Methods

### Search strategy

**Fig. 1:**
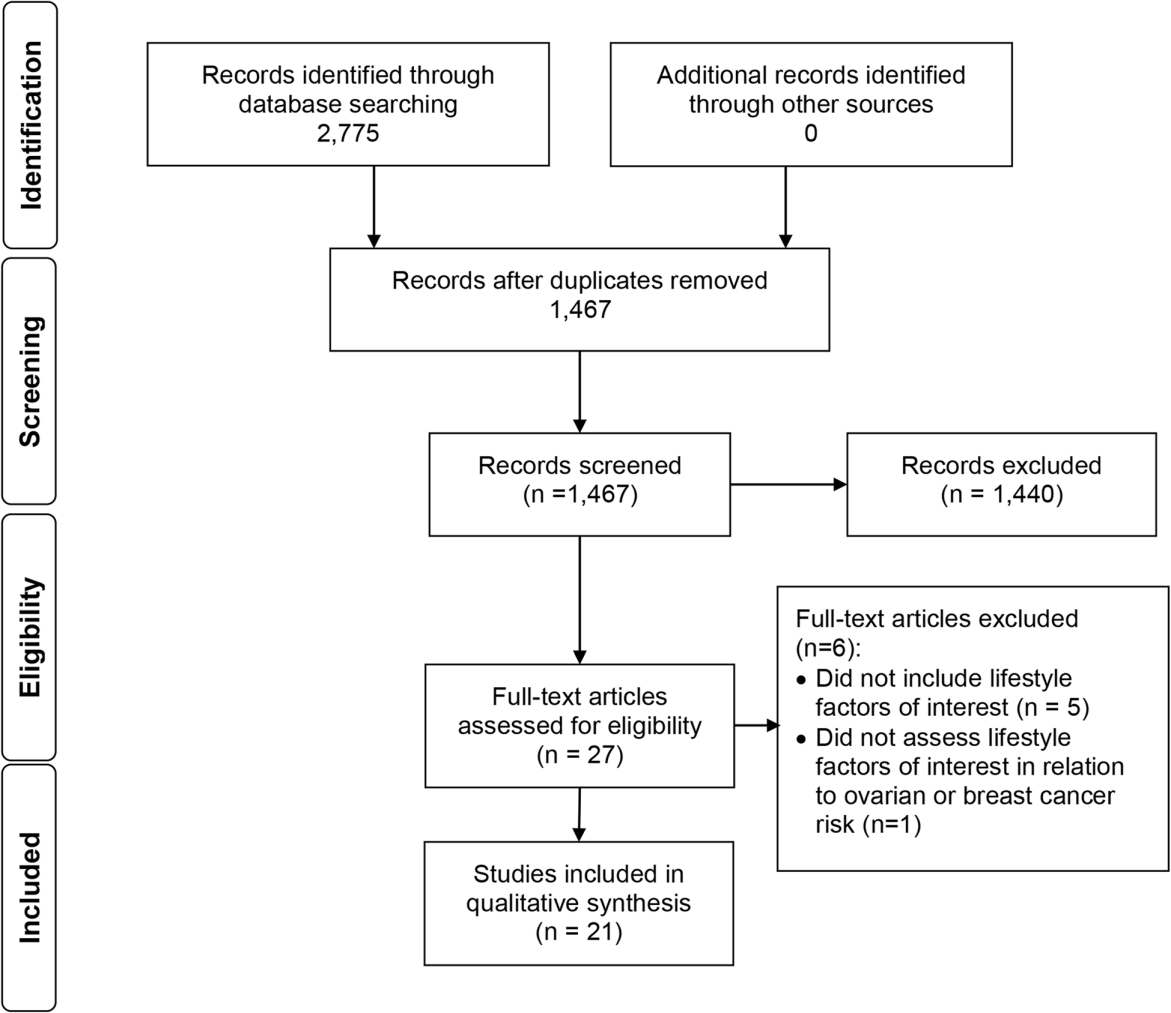
PRISMA Flow Diagram

The search terms and search strategy were developed by four authors (AMC, LG, KBE, KJK), one of whom (KJK) is a medical research librarian specializing in systematic reviews. A systematic search was performed in MEDLINE, EMBASE, Cochrane Library, and ClinicalTrials.gov from inception to October 3, 2019. Search structures, subject headings, and keywords were tailored to each database by KJK. The search was expanded through citation chaining (forward and backward) of included studies. The search terms used can be found in the MEDLINE search strategy [see Additional File 1]. Findings are reported in accordance with the Preferred Reporting Items for Systematic Reviews and Meta-Analyses (PRISMA) checklist [8](Fig. 1). The protocol is registered in PROSPERO(ID:CRD42017060007).

### Review process, selection Criteria & Data Extraction

Two authors (AMC, LG) independently screened the titles and abstracts of the articles to identify potentially relevant studies. Studies that passed the title/abstract review were retrieved for full-text review. Disagreements were resolved by consensus and by seeking the opinion of a third author (KBE). Inclusion criteria consisted of studies that: included individuals with *BRCA1/2* pathogenic germline gene variants; evaluated weight status, weight change, dietary habits (as defined by dietary patterns, food and beverage intake, multivitamin and mineral supplementation), or physical activity in relation to ovarian or breast cancer risk; published in English; and included human subjects only.

### Risk-of-Bias assessment

The Quality Assessment Tool for Quantitative Studies, version 2010, was used for risk-of-bias assessment [9, 10]. Six components were evaluated to determine overall study quality: selection bias, design, confounders, blinding, data collection method, withdrawals/dropouts. Quality scores were assigned per criteria described elsewhere [10].

### Analysis

Qualitative synthesis of data is provided in narrative form. A meta-analysis was not conducted due to the limited number of studies and heterogeneity in study design and outcome measures.

## Results

### Risk-of-Bias summary

Table 1 provides details of the risk of bias assessment for all studies. Four studies received an overall quality score of strong [11–14], 16 received moderate [15–29], and one received weak [30].

**Table 1:** Risk of Bias Summary

Cancer Type	Author, Year	Selection Bias	Study Design	Confounders	Blinding	Data Collection Method	Withdrawals & Dropouts	Quality Score
Ovarian Cancer	Gronwald J et al., 2006	Strong	Moderate	Weak	N/A	Weak	N/A	Weak
	McGee J et al., 2012	Weak	Moderate	Strong	N/A	Moderate	N/A	Moderate
	Qian F et al., 2019	Strong	Moderate	Strong	N/A	Weak	N/A	Moderate
	Abbas S et al., 2019	Strong	Moderate	Weak	N/A	Strong	N/A	Moderate
Breast Cancer	Cybulski C et al., 2015	Strong	Moderate	Strong	N/A	Weak	N/A	Moderate
	Dennis J et al., 2010	Strong	Moderate	Strong	N/A	Moderate	N/A	Moderate
	Dennis J et al., 2011	Strong	Moderate	Strong	N/A	Strong	N/A	Strong
	Gronwald J et al., 2006	Strong	Moderate	Weak	N/A	Weak	N/A	Weak
	Kim SJ et al., 2019	Strong	Moderate	Strong	N/A	Moderate	N/A	Moderate
	King MC et al., 2003	Strong	Moderate	Moderate	N/A	Weak	N/A	Moderate
	Ko KP et al., 2013	Strong	Moderate	Strong	N/A	Strong	N/A	Strong
	Kotsopoulos J et al., 2005	Moderate	Moderate	Strong	N/A	Moderate	N/A	Moderate
	Lammert J et al., 2018	Strong	Moderate	Strong	N/A	Strong	N/A	Strong
	Lecarpentier J et al., 2011	Strong	Moderate	Strong	N/A	Moderate	N/A	Moderate
	Manders P et al., 2011	Strong	Moderate	Strong	N/A	Weak	N/A	Moderate
	McGuire V et al., 2006	Moderate	Moderate	Strong	N/A	Moderate	N/A	Moderate
	Moorman PG et al., 2010	Strong	Moderate	Weak	N/A	Moderate	N/A	Moderate
	Nkondjock A, Ghadirian P, et al., 2006	Strong	Moderate	Strong	N/A	Moderate	N/A	Moderate
	Nkondjock A, Robidoux A et al., 2006	Moderate	Moderate	Strong	N/A	Strong	N/A	Moderate
	Nkondjock A et al., 2007	Strong	Moderate	Strong	N/A	Strong	N/A	Strong
	Pijpe A et al., 2010	Moderate	Moderate	Strong	N/A	Moderate	N/A	Moderate
	Qian F et al., 2019	Strong	Moderate	Strong	N/A	Weak	N/A	Moderate

N/A = Not Applicable due to study design

### Ovarian Cancer risk

Table 2 provides study characteristics and results of the studies reviewed for both ovarian and breast cancer. Gronwald and colleagues [30] case-control study did not observe a significant association between coffee consumption and ovarian cancer risk (OR 0.7, 95%CI 0.4,1.3) among 348 women with *BRCA1* pathogenic germline gene variant [30]. Information regarding quantity of coffee consumed or statistical adjustments was not provided. No studies assessed PA.

**Table 2:** Ovarian & Breast Cancer Risk Study Characteristics and Results

Ovarian Cancer Risk					
Author, Year	Patient Characteristics	Study Design, Data Source	Lifestyle Factor	Measurement Method	Results
Gronwald J et al., 2006	348 matched case-control pairs of women with *BRCA1* pathogenic germline gene variant	Case-control, International Hereditary Cancer Center in Szczecin or elsewhere in Poland	Dietary habits- coffee	Standardized questionnaire that inquired about reproductive and medical history, smoking history, oral contraceptive use, and coffee consumption.	Coffee consumption and ovarian cancer risk: OR, 0.7 (95%CI 0.4,1.3) Data related to other factors (i.e.- reproductive history, oral contraceptive use, smoking history) and ovarian cancer risk available in paper. *Statistical adjustments not specified*
McGee J et al., 2012	469 matched case-control pairs of women with *BRCA1* and *BRCA2* pathogenic germline gene variants 403 pairs of women with *BRCA1* pathogenic germline gene variant 66 pairs of women with *BRCA2* pathogenic germline gene variant	Case-control, data from 50 participating centers	Weight status, weight change	Standardized questionnaire that inquired about reproductive and medical history, smoking history, oral contraceptive use, and the following questions related to weight and weight history: weight at age 18, 30, 40, and current weight and height.	No significant associations were observed between weight status/weight change variables and ovarian cancer risk. No significant differences were observed between cases and controls for the following weight status/weight change variables: height, current weight, weight at ages 18, 30, and 40; changes in weight from ages 18–30, 30–40, 18–40; and BMI at ages 18, 30, and 40*. *Data adjusted for age at menarche, parity, oral contraceptive use, height, and history of hormone replacement therapy
Qian F et al., 2019	7516 women with BMI data 2923 ovarian cancer cases 2319 *BRCA1* 604 *BRCA2* pathogenic germline gene variants Total sample size for Consortium of Investigators of Modifiers of BRCA1/2 (CIMBA): 22,588 women with *BRCA1* or *BRCA2* pathogenic germline gene variant 14,676 *BRCA1* − 7360 women with breast cancer (cases) 7912 *BRCA2* − 4091 cases	Case-Control, data from CIMBA−33 countries including 55 centers	Weight status	Questionnaire of self-reported height and weight to calculate observed BMI at date of questionnaire and during young adulthood Included Mendelian Randomization approach: Calculated weighted genetic score for BMI and height (see paper for details)	Observed BMI and Ovarian Cancer Risk at Date of Questionnaire: Per 5 kg/m^2^ (participants/number of events): -All participants (6964/715): HR, 1.04 (0.94, 1.14)* -*BRCA1* (4401/543): HR, 1.06 (0.95, 1.17)** -*BRCA2* (3115/229): HR, 0.96 (0.81, 1.15)** -Premenopausal (7516/102): HR, 1.25 (1.06, 1.48)*** -Postmenopausal (4257/670): HR, 0.98 (0.88, 1.10)*** -Serous (7223/312): HR, 0.98 (0.84, 1.15)**** -Non-serous (7223/167): HR, 1.25 (1.06, 1.49)**** Observed BMI and Ovarian Cancer Risk in Young Adulthood: Per 5 kg/m^2^ (participants/number of events): -All participants (5210/516): HR, 0.92 (0.74, 1.14)* -*BRCA1* (3134/380): HR, 0.92 (0.71, 1.18)** -*BRCA2* (2283/156): HR, 1.00 (0.74, 1.36)** -Premenopausal (5417/67): HR, 1.34 (0.97, 1.84)*** -Postmenopausal (3094/469): HR, 0.82 (0.65, 1.04)*** BMI Genetic Score and Ovarian Cancer Risk at Date of Questionnaire: Per 5 kg/m^2^ (participants/number of events): -All participants (22,588/2923): HR, 1.10 (0.86, 1.42)**** -*BRCA1* (14,676/2319): HR, 1.16 (0.78, 1.53)** -*BRCA2* (7912/604): HR, 0.81 (0.46, 1.43)** -Premenopausal (22,588/967): HR, 1.59 (1.08, 2.33)*** -Postmenopausal (9219/1955): HR, 0.80 (0.58, 1.11)*** -Serous (20,978/892): HR, 0.92 (0.59, 1.43)**** -Non-serous (20,978/421): HR, 1.60 (0.83, 3.08)**** *Data from fully adjusted model. Adjusted for principal components, country, birth cohort, mutation status, menopausal status, parity and age at menarche. **Data adjusted for principal components, birth cohort, country of enrollment, menopausal status in a weighted cox model. ***Data adjusted for principal components, birth cohort, country of enrollment, mutation status. ****Data adjusted for principal components, birth cohort, country of enrollment, menopausal status, mutation status. Data for other multivariable adjustments and height are available in the paper.
**Breast Cancer Risk**					
**Author, Year**	**Patient Characteristics**	**Study Design, Data Source**	**Lifestyle Factor**	**Measurement Method**	**Results**
Cybulski C et al., 2015	3067 women with *BRCA* pathogenic germline gene variants 2498 *BRCA1* 569 *BRCA2*	Prospective cohort, data from 78 participating centers in 12 countries Average 5.4-year follow-up	Dietary habits- alcohol	Standardized questionnaire including questions related to family and personal history of cancer, medical and reproductive history, and the following questions related to alcohol consumption: Current consumption, age at first and last use, average number of drinks per week, type of alcohol consumed. Baseline questionnaire completed at time of clinic appointment and follow-up questionnaires completed every 2 years thereafter	259 incident cases observed. Significant relationships were not observed between breast cancer risk and the following alcohol variables in adjusted models*: ever use of alcohol, cumulative consumption, age at first use, alcohol use by the first full-term birth. Significant relationships were not observed between ever or current use of alcohol and breast cancer risk by menopausal status, pathogenic gene variant, and age of breast cancer diagnosis among cases. *Data adjusted for age at baseline, BRCA1/2 pathogenic germline gene variant, age at menarche, oral contraceptive use, history of breast feeding, mean parity, oophorectomy status, and country of residence.
Dennis J et al., 2010	1925 matched case-control pairs of women with *BRCA1/2* pathogenic germline gene variants 1480 *BRCA1* 445 *BRCA2*	Case-Control, data from 54 centers in 8 countries	Dietary habits- alcohol	Standardized questionnaire with questions related to alcohol consumption: if consume alcohol, number of drinks per week.	Drinks consumed per week and breast cancer risk in women with*BRCA1*pathogenic germline gene variants*: *BRCA1* --none**: 1.00 --0-3: OR, 0.77 (0.67,0.94) --4-9: OR, 0.98 (0.73,1.32) -- ≥ 10: OR, 0.55 (0.33,0.91) *p-trend = 0.03* Type of alcohol consumed per week and breast cancer risk among women with*BRCA1*pathogenic germline gene variants*: exclusive wine consumers --none**: 1.00 --0-3: OR, 0.62 (0.45,0.87) --4-9: OR, 0.82 (0.41,1.67) -- ≥ 10: OR, 0.39 (0.11,1.45) *p-trend = 0.01* other alcohol types (beer and spirits) --none**: 1.00 --0-3: OR, 0.62 (0.43,0.91) --4-9: OR, 1.07 (0.40,2.85) -- ≥ 10: OR, 0.70 (0.13,3.75) *p-trend = 0.01* Significant associations not observed in women with *BRCA2* pathogenic germline gene variants for any alcohol variables. *Data adjusted for ethnicity, menopause, oral contraceptive use, hormone-replacement therapy use, smoking status, history of oophorectomy, BMI, and parity. **Individuals who reported that they did not currently consume alcoholic beverages
Dennis J et al., 2011	857 breast cancer cases diagnosed within the last 10 years of data collection 10 cases with *BRCA1* pathogenic germline gene variant 33 cases with *BRCA2* pathogenic germline gene variant 814 cases without *BRCA* pathogenic germline gene variant	Case-only, data from Centre Hospitalier de L’Universite de Montreal	Dietary habits- alcohol	Interviewer administered food frequency questionnaire developed by the NCI of Canada. Questionnaire inquired about alcohol consumption in the year prior to breast cancer diagnosis Also completed questionnaire related to other lifestyle factors: ethnicity, family history, reproductive and medical history, menopausal status, smoking habits, oral contraceptive use, hormone replacement therapy use (data not shown in this table)	Average time between diagnosis and interview was 3.1 years. 10/10 (100%) women with *BRCA1* pathogenic germline gene variants consumed alcohol within the year prior to breast cancer diagnosis. 30/33 (90.9%) women with *BRCA2* pathogenic germline gene variants consumed alcohol within the year prior to breast cancer diagnosis. Alcohol consumption among women with breast cancer and*BRCA1/2*pathogenic germline gene variants compared to women with breast cancer without*BRCA1/2*pathogenic germline gene variant*^,^**: *BRCA1* --total alcohol (3 drinks/week): COR, 0.79 (0.22,2.83) --wine (2 drinks/week): COR, 0.38 (0.08,1.81) --other alcohol (0.33 drinks/week): COR, 2.49 (0.64,9.73) *BRCA2* --total alcohol (3 drinks/week): COR, 1.99 (0.96,4.11) --wine (2 drinks/week): COR, 1.58 (0.78,3.17) --other alcohol (0.33 drinks/week): COR, 2.15 (1.03,4.50) *Data adjusted for age at diagnosis **Drinks per week dichotomized by case median
Lecarpentier J et al., 2011	1337 women with *BRCA* pathogenic germline gene variants 499 women with breast cancer and *BRCA* pathogenic germline gene variant − 332 *BRCA1* −167 *BRCA2* 838 women without breast cancer but with *BRCA* pathogenic germline gene variant − 531 *BRCA1* −307 *BRCA2*	Case-Control, data from French National *BRCA 1/2* Carrier Cohort (GENEPSO)	Dietary habits- alcohol	Standardized questionnaire administered by mail inquiring about reproductive factors, tobacco use, alcohol consumption at age 20, and history of chest x-ray exposure	Among women with*BRCA1*pathogenic germline gene variant*: - When alcohol use was stratified by tobacco use (ever vs never smoker) there were no significant interactions observed (*p* > 0.05). -When tobacco use was stratified by alcohol use (ever vs never use of alcohol) the only significant interactions observed were among women who reported never drinking alcohol. Among women with*BRCA2*pathogenic germline gene variant*: Ever use --No: 1.00 *--*Yes: HR, 1.21 (0.68,2.15) Consumed > 5 glasses per week at age 20 --No: 1.00 --Yes: HR, 1.78 (0.97,3.27) -There were no significant interactions between alcohol and tobacco use (*p* = 0.75). Therefore, analysis for tobacco and alcohol use were not stratified among women with *BRCA2* pathogenic germline gene variants as it was for women with *BRCA1* pathogenic germline gene variant. *Data adjusted for parity, menopausal status, gene, smoking history, number of years of smoking interruption
McGuire V et al., 2006	804 women with *BRCA* pathogenic germline gene variants 323 women with breast cancer −195 *BRCA1* − 128 *BRCA2* 481 women without breast cancer − 302 *BRCA 1* − 179 *BRCA 2*	Case-Control, data from six research institutions in USA, Canada, and Australia who were part of Breast Cancer Family Registry, and both the Kathleen Cuningham Foundation Consortium for Research into Familial Breast Cancer in Australia, and the Ontario Cancer Genetics Network in Canada	Dietary habits- alcohol	Risk factor questionnaire including questions related to alcohol consumption	Alcohol consumption and breast cancer risk in women with the*BRCA1*pathogenic germline gene variant*,**: Ever use --No: 1.00 --Yes: OR, 1.06 (0.73,1.52) Current use --No: 1.00 --Yes: OR, 0.96 (0.67,1.37) Years of drinking --Nonusers: 1.00 --1-29: OR, 1.07 (0.64,1.76) -- > 29: OR, 0.93 (0.62,1.39) --Trend per 10 years of drinking: OR, 0.98 (*p* = 0.5) Daily alcohol intake (g/d) --Nonusers: 1.00 --1-4: OR, 0.63 (0.34,1.18) -- > 4: OR, 1.14 (0.77,1.69) --Trend per 10 g: OR, 1.02 (*p* = 0.4) Alcohol consumption and breast cancer risk in women with the*BRCA2*pathogenic germline gene variant*,**: Ever use --No: 1.00 --Yes: OR, 0.66 (0.45,0.97) Current use --No: 1.00 --Yes: OR, 1.11 (0.76,1.63) Years of drinking --Nonusers: 1.00 --1-29: OR, 0.40 (0.21,1.74) -- > 29: OR, 0.89 (0.59,1.34) --Trend per 10 years of drinking: OR, 1.02 (p = 0.4) Daily alcohol intake (g/d) --Nonusers: 1.00 --1-4: OR, 0.41 (0.22,0.77) -- > 4: OR, 0.79 (0.52,1.18) --Trend per 10 g: OR, 1.00 (*p* = 0.9) Significant differences in breast cancer risk was not observed by alcohol type (i.e. wine, beer, liquor). *Data adjusted for age (as a continuous variable), family history (number of first degree relatives with history of breast or ovarian cancer), smoking status, and number of full-term pregnancies. **Stratified by age (< 40 years and > 40–49 years) and study sites.
Moorman PG et al., 2010	1381 female breast cancer cases 283 women with breast cancer and *BRCA 1* pathogenic germline gene variant 204 women with breast cancer and *BRCA 2* pathogenic germline gene variant 894 sporadic breast cancer cases	Case-Only, data from the Genetic and Environmental Modifiers of *BRCA 1* and *BRCA 2* pathogenic germline gene variants Study (GEMS) Cases were identified either prospectively or retrospectively pending on which center was collecting data	Dietary habits- alcohol Weight status	Risk factor questionnaire that inquired about demographic information, medical and reproductive history, use of oral contraceptives, smoking status, alcohol use, and weight history Prospective enrollment of breast cancer cases used either the GEMS questionnaire or included a supplement to a pre-existing questionnaire to capture lifestyle data not included in the original questionnaire but included in the GEMS questionnaire Retrospective enrollment of breast cancer cases used a similar risk factor questionnaire but not the specific GEMS questionnaire	Alcohol use and breast cancer risk in women with breast cancer and*BRCA1*pathogenic germline gene variant compared to women with breast cancer without*BRCA1*pathogenic germline gene variant*: Never use: 1.00 Ever use: IRR, 0.65 (0.48,0.90) Weight history and breast cancer risk in women with breast cancer and*BRCA1*pathogenic germline gene variant compared to women with breast cancer without*BRCA1*pathogenic germline gene variant*: BMI (kg/m^2^) one year before diagnosis -- < 25: IRR, 1.00 -- ≥ 25 to < 30: IRR, 0.99 (0.65,1.51) -- ≥ 30: IRR, 1.38 (0.89,2.13) BMI (kg/m^2^) at age 18 -- < 25: IRR, 1.00 -- ≥ 25 to < 30: IRR, 0.76 (0.48,1.20) -- ≥ 30: IRR, 1.15 (0.68,1.94) Alcohol use and breast cancer risk in women with breast cancer and*BRCA2*pathogenic germline gene variant compared to women with breast cancer without*BRCA2*pathogenic germline gene variant*: Never use: 1.00 Ever use: IRR, 0.80 (0.55,1.16) Weight history and breast cancer risk in women with breast cancer and*BRCA2*pathogenic germline gene variant compared to women with breast cancer without*BRCA2*pathogenic germline gene variant*: BMI (kg/m^2^) one year before diagnosis -- < 25: IRR, 1.00 -- ≥ 25 to < 30: IRR, 1.09 (0.70,1.70) -- ≥ 30: IRR, 1.15 (0.67,1.90) BMI (kg/m^2^) at age 18 -- < 25: IRR, 1.00 -- ≥ 25 to < 30: IRR, 0.81 (0.50,1.31) -- ≥ 30: IRR, 0.68 (0.33,1.38) *Data adjusted for age and center.
Gronwald J et al., 2006	348 matched case-control pairs with *BRCA1* pathogenic germline gene variant	Case-control, data from International Hereditary Cancer Center in Szczecin or elsewhere in Poland	Dietary habits- coffee	Standardized questionnaire that inquired about reproductive and medical history, smoking history, oral contraceptive use, and coffee consumption.	No associations observed with breast cancer risk Data related to other factors (i.e.- reproductive history, oral contraceptive use, smoking history) and breast cancer risk available in paper. *Statistical adjustments not specified*
Nkondjock A, Ghadirian P, et al., 2006	845 matched case-control pairs 652 pairs with *BRCA1* 193 pairs with *BRCA2* Cases were diagnosed with breast cancer as their first or only cancer	Case-Control, data from 40 centers in 4 countries	Dietary habits- coffee	Standardized questionnaire that inquired about demographic information, ethnicity, parity, family history, reproductive and medical history, use of oral contraceptives, smoking history, alcohol consumption and coffee consumption. -questions related to caffeinated and decaffeinated coffee consumption include: ever use, current use, age when started drinking coffee, age when stopped drinking coffee, average daily coffee consumption 7.8 years on average elapsed from diagnosis date to questionnaire administration	Caffeinated coffee consumption and breast cancer risk*: --0 cups/day: 1.00 --1-3 cups/day: OR, 0.90 (0.72,1.12) --4-5 cups/day: OR, 0.75 (0.47,1.19) -- ≥ 6 cups/day: OR, 0.31 (0.13,0.71) *p-trend = 0.02* Decaffeinated coffee consumption and breast cancer risk*: --0 cups/day: 1.00 --1-3 cups/day: OR, 0.99 (0.72,1.36) --4-5 cups/day: OR, 1.14 (0.30,4.31) -- ≥ 6 cups/day: NA *p-trend = 1.00* Total coffee consumption (caffeinated + decaffeinated) and breast cancer risk*: --0 cups/day: 1.00 --1-3 cups/day: OR, 0.89 (0.70,1.13) --4-5 cups/day: OR, 0.73 (0.48,1.10) -- ≥ 6 cups/day: OR, 0.51 (0.26,0.98) *p-trend = 0.03* Caffeinated coffee consumption and breast cancer risk by*BRCA1/2*pathogenic germline gene variants*: *BRCA1* --0 cups/day: 1.00 --1-3 cups/day: OR, 0.82 (0.64,1.06) --4-5 cups/day: OR, 0.67 (0.39,1.16) -- ≥ 6 cups/day: OR, 0.25 (0.09,0.71) *p-trend = 0.009* *BRCA2* --0 cups/day: 1.00 --1-3 cups/day: OR, 1.26 (0.78,2.08) --4-5 cups/day: OR, 1.17 (0.48,2.83) -- ≥ 6 cups/day: OR, 0.40 (0.09,1.73) *p-trend = 0.84* *Data adjusted for parity, smoking status, oral contraceptive use, alcohol consumption and BMI at age 30.
Ko KP et al., 2013	491 women with *BRCA1/2* pathogenic germline gene variant 370 cases with breast cancer 1789 women without pathogenic germline gene variants − 1632 cases with breast cancer *Included in an analysis of all breast cancer cases regardless of pathogenic germline gene variant status* *(this data is not reported in this table)*	Retrospective cohort, data from KOHBRA (Korean Hereditary Breast Cancer Study)	Dietary habits- food intake	Validated food frequency questionnaire developed by the Korean National Institutes of Health	Dietary intake divided into quartiles. Intake of the following food items was assessed: vegetables, fruit, meat, seafood, soybean products. Significant associations were not observed in women with *BRCA1/2* pathogenic gene variants for vegetable, fruit and seafood intake in and breast cancer risk. Meat and soybean product intake and breast cancer risk among women with*BRCA1/2*pathogenic germline gene variants carriers combined*: Meat (number of food items) --Q1 (0): 1.00 --Q2 (1): HR, 1.03 (0.64,1.68) --Q3 (2): HR, 1.29 (0.77,2.17) --Q4 (3–10): HR, 1.97 (1.13, 3.44) *p-trend = 0.026* Soybean products (number of food items) --Q1 (0–1): 1.00 --Q2 (2): HR, 1.09 (0.68,1.76) --Q3 (3): HR, 0.72 (0.45,1.14) --Q4 (4–5): HR, 0.39 (0.19, 0.79) *p-trend = 0.005* Meat and soybean product intake and breast cancer risk among women with*BRCA2*pathogenic germline gene variant*: Meat (number of food items) --Q1 (0): 1.00 --Q2 (1): HR, 0.83 (0.42,1.64) --Q3 (2): HR, 1.16 (0.57,2.37) --Q4 (3–10): HR, 2.48 (1.26, 4.89) *p-trend = 0.027* Soybean products (number of food items) --Q1 (0–1): 1.00 --Q2 (2): HR, 1.41 (0.75,2.65) --Q3 (3): HR, 0.76 (0.40,1.44) --Q4 (4–5): HR, 0.38 (0.16, 0.93) *p-trend = 0.005* Significant associations were not observed in women with *BRCA1* pathogenic germline gene variant for meat and soybean intake and breast cancer risk *Data adjusted for menarche, caloric intake, years of education, smoking history, alcohol intake history, exercise habits, and parity.
Nkondjock A and Ghadirian P, 2007	89 cases with *BRCA1* or *BRCA2* pathogenic germline gene variants 48 controls with *BRCA1* or *BRCA2* pathogenic germline gene variants 46 controls who did not have *BRCA1/2* pathogenic germline gene variants	Case-Control, data from 80 French Canadian families	Dietary habits- diet quality	Validated food frequency questionnaire developed by the National Cancer Institute of Canada. The questionnaire covered the 1-year period prior to diagnosis for cases and the corresponding time period for controls. Included dietary habits, multivitamins, supplements and alcohol use. Assessed dietary intake via the following diet-quality indexes: -Alternative healthy eating index (AHEI) -Diet quality index-revised (DQI-R) -alternate Mediterranean diet index (aMED) -Canadian healthy eating index (CHEI) Also included general lifestyle questionnaire to collect baseline data on other lifestyle variables. Logistic regression analysis was only conducted on diet quality variables.	The only significant differences between cases and controls were among the following variables (*p* < 0.05): total energy intake (kcal/d) --Cases: 2589 ± 1142 --Controls- *BRCA 1/2* carriers: 2167 ± 830 --Controls- non-carriers: 2146 ± 720 age at maximum weight (years) --Cases: 46.2 ± 13.3 --Controls- *BRCA 1/2* carriers: 38.8 ± 13.3 --Controls- non-carriers: 41.5 ± 16.5 maximum BMI (kg/m^2^) --Cases: 27.4 ± 5.6 --Controls- *BRCA 1/2* carriers: 25.0 ± 4.5 --Controls- non-carriers: 26.8 ± 6.1 Diet quality and breast cancer risk when comparing*BRCA1/2*pathogenic germline gene variants cases and controls*: DQI-R --Q1: 1.00 --Q2: OR, 1.04 (0.43,2.52) --Q3: OR, 0.35 (0.12,1.02) *p-trend = 0.034* CHEI --Q1: 1.00 --Q2: OR, 0.42 (0.15,1.18) --Q3: OR, 0.18 (0.05,0.68) p-trend = 0.006 Diet quality and breast cancer risk when comparing*BRCA1/2*pathogenic germline gene variant cases and non-carrier controls*: DQI-R --Q1: 1.00 --Q2: OR, 0.54 (0.21,1.36) --Q3: OR, 0.21 (0.07,0.62) *p-trend = 0.003* *Data adjusted for age, physical activity and total energy intake.
Kim SJ et al., 2019	400 women with *BRCA1/2* pathogenic germline gene variant 129 cases with breast cancer	Case-Control, data from 10 different centers in Canada	Dietary habits- nutrient intake Folic acid B6 B12	Open-ended questionnaire collecting the following information about each supplement taken since age 18: -type of supplement -brand name of supplement -weekly frequency of supplement use -supplement dose -duration of use	The following supplements were not significantly associated with breast cancer risk in adjusted models (with never use as the reference): -Multivitamin, ever use -Folic acid, ever use -B6, 0.02 - ≤ 0.20 mg/d or > 0.20 mg/d Ever use of prenatal supplement (with never use as reference): OR, 0.57 (0.34, 0.95)* OR, 0.60 (0.35, 1.02)** Any folic-acid containing supplement (with never use as reference): OR, 0.81 (0.50, 1.29)* OR, 0.45 (0.25, 0.79)** Total daily average of folic acid (with never use as reference): 8.56 - ≤ 89.29 mcg/d OR, 0.39 (0.19, 0.81)** > 89.29 mcg/d OR, 0.54 (0.27, 1.10)** Total daily average of B12 (with never use as reference): 0.02 - ≤ 0.34 mcg/d OR, 0.48 (0.24, 0.96)** > 0.34 mcg/d OR, 0.61 (0.33, 1.12)** Ever use of any folic-acid containing supplement, when assessed by *BRCA* mutation, revealed a significant association with breast cancer risk among women with *BRCA1* pathogenic germline gene variants*:* OR, 0.30 (0.14,0.65)** Ever use of any folic-acid containing supplement assessed by parity did not reveal significant association with breast cancer risk. *Data adjusted for age and BRCA1/2 pathogenic germline gene variant **Data adjusted for age, BRCA1/2 pathogenic germline gene variant, BMI, parity, alcohol consumption, smoking status
Nkondjock A, Robidoux A, et al., 2006	89 cases with *BRCA1* or *BRCA2* pathogenic germline gene variants 48 controls with *BRCA1* or *BRCA2* pathogenic germline gene variants	Case-Control, data from 80 French Canadian families	Dietary habits- nutrient intake Weight change Physical activity	Validated semi-quantitative food frequency questionnaire that covered the 1-year period prior to diagnosis for cases and the corresponding time period for controls Lifestyle core questionnaire for physical activity, weight change, and other lifestyle factors such as smoking history, menopausal status, oral contraceptive use, medical and reproductive history Physical activity information covered the 2-year period before diagnosis or interview for controls Weight history information included height, current weight, weight at age 18 and 30.	Energy intake and breast cancer risk: Total energy intake (kcal/d)* --Q1 ≤ 1724: 1.00 --Q2 > 1724 and ≤ 2339: OR, 1.17 (0.44,3.13) --Q3 > 2339: OR, 2.76 (1.10,7.02) *p-trend = 0.026* Significant associations were not observed for intake of the following in adjusted models: fat, protein, carbohydrates, poly-unsaturated fatty acids, mono-unsaturated fatty acids, saturated fatty acids, alcohol, beer, wine, spirits, vitamins C and E, fiber, folate, caffeine. Weight change and breast cancer risk**: Age at maximum BMI (years) --Q1 ≤ 34: 1.00 --Q2 > 34 and ≤ 43: OR, 1.12 (0.41,3.05) --Q3 > 43: OR, 2.90 (1.01,8.36) *p-trend = 0.043* Weight gain since age 18 (pounds) --Q1 ≤ 12: 1.00 --Q2 > 12 and ≤ 35: OR, 3.63 (1.18,11.22) --Q3 > 35: OR, 4.64 (1.52,14.12) *p-trend = 0.011* Weight gain since age 30 (pounds) --Q1 ≤ 8: 1.00 --Q2 > 8 and ≤ 20: OR, 3.43(1.16,10.14) --Q3 > 20: OR, 4.11 (1.46,11.56) *p-trend = 0.013* No significant association was observed between physical activity variables (i.e. weekly MET hours of moderate activity, weekly MET hours of vigorous activity, total weekly MET hours of physical activity) and breast cancer risk. *Data adjusted for age, maximum lifetime BMI and physical activity **Data adjusted for age, physical activity and total energy intake.
Abbas S et al., 2019	200 samples from women with *BRCA1* rs80356932 & *BRCA2* rs80359182 pathogenic germline gene variants 100 samples from women with breast cancer	Case-control, data from three hospitals in Pakistan: Jinnah Hospital, Fauji Foundation Hospital, and INMOL Hospital Lahore	Weight Status	BMI extracted from medical record	Breast cancer was most prevalent in women with obesity, per BMI (*p* = 0.002) Normal weight (BMI 18.5–24.9 kg/m^2^) as reference Underweight (BMI < 18.5 kg/m^2^): OR, 1.71 (0.41, 7.00) Overweight (BMI 25.0–29.9 kg/m^2^): OR, 3.06 (1.36, 6.87) Obese (BMI > 30 kg/m^2^): OR, 4.09 (1.91, 8.75)
Kotsopoulos J et al., 2005	1073 matched case-control pairs 797 pairs with *BRCA1* pathogenic germline gene variants 276 pairs with *BRCA2* pathogenic germline gene variants Cases were diagnosed with breast cancer as their first or only cancer	Case-Control, data from 41 centers in 5 countries with research protocols including *BRCA* pathogenic germline gene variant status	Weight change	Standardized questionnaire that inquired about demographic information, ethnicity, parity, family history, reproductive and medical history, use of oral contraceptives, smoking history, weight at birth, age 18, 30 and 40, current weight and height 8.8 years on average elapsed from diagnosis date to questionnaire administration	Weight change between 18 and 30 years: --loss of ≥10#: OR, 0.66 (0.46,0.93) --loss of < 10# to gain of ≤10#: OR, 1.00 --gain of 10 to ≤20#: OR, 1.19 (0.96,1.49) --gain of > 20#: OR, 1.00 (0.77,1.30) *p-trend = 0.46* Weight change between 18 and 30 years by case subjects’ age at diagnosis: > 30 to ≤40 years --loss of ≥10#: OR, 0.47 (0.28,0.79) --loss of < 10# to gain of ≤10#: OR, 1.00 --gain of 10 to ≤20#: OR, 1.25 (0.91,1.71) --gain of > 20#: OR, 1.03 (0.72,1.47) *p-trend = 0.48* > 40 years --loss of ≥10#: OR, 0.97 (0.52,1.65) --loss of < 10# to gain of ≤10#: OR, 1.00 --gain of 10 to ≤20#: OR, 1.16 (0.85,1.59) --gain of > 20#: OR, 0.95 (0.64,1.43) *p-trend = 0.75* Weight change between 18 and 30 years by*BRCA1/2*pathogenic germline gene variant: *BRCA 1* --loss of ≥10#: OR, 0.35 (0.18,0.67) --loss of < 10# to gain of ≤10#: OR, 1.00 --gain of 10 to ≤20#: OR, 1.29 (0.91,1.83) --gain of > 20#: OR, 1.09 (0.73,1.62) *p-trend = 0.34* *BRCA2* --loss of ≥10#: OR, 0.88 (0.35,2.23) --loss of < 10# to gain of ≤10#: OR, 1.00 --gain of 10 to ≤20#: OR, 1.08 (0.50,2.35) --gain of > 20#: OR, 0.77 (0.33,1.81) *p-trend = 0.70* When assessed by pathogenic gene variant and parity (0, 1, ≥2), with loss of < 10# between age 18 and 30 years as the reference group, the only significant association observed was with gain of > 10# among women with*BRCA 1*pathogenic germline gene variant with parity ≥2, OR, 1.44 (1.01,2.04) *Only univariate results reported in paper. Per authors, results from analyses adjusted for oral contraceptive use, smoking, oophorectomy, and parity were similar to univariate results.*
Manders P et al., 2011	558 women with *BRCA 1* pathogenic germline gene variants 167 women with *BRCA 2* pathogenic germline gene variants 218 women diagnosed with breast cancer within the 10-year period of questionnaire − 170 *BRCA 1* − 48 *BRCA 2*	Retrospective Cohort, data from HEBON study (Hereditary Breast and Ovarian Cancer Study, the Netherlands)	Weight status/ weight change	Standardized risk factor questionnaire Questions related to body weight/weight change include weight at age 18, current weight and current height, body weight in different age periods (10-year increments starting at age 20 up to 70+) Specifically assessed weight change in relation to menopausal status	Significant associations were not observed among the following variables in relation to premenopausal breast cancer risk among women with *BRCA 1/2* pathogenic germline gene variants: body weight at age 18, BMI at age 18, current body weight, current BMI, adult weight change, and relative weight change*^,^**. Current weight (kg) and postmenopausal breast cancer risk*^,^***: < 72: 1.00 ≥72: HR, 2.10 (1.23,3.59) -No other significant associations observed for weight change variables and postmenopausal breast cancer risk. *Data analyzed as time-varying Cox-proportional hazards model, stratified by gene and birth cohort, clustered for family, and adjusted for parity, type of menopause and history of hormone replacement therapy, and lifetime sports activity. **Results observed were the same for weighted cohort approach analysis and unweighted analysis. ***Results are for unweighted analysis, underpowered to conduct weighted cohort approach analysis.
Qian F et al., 2019	7516 women with BMI data − 4401 *BRCA1* − 3115 *BRCA2* Total sample size for Consortium of Investigators of Modifiers of BRCA1/2 (CIMBA): 22,588 women with *BRCA1* or *BRCA2* pathogenic germline gene variant 14,676 *BRCA1* − 7360 women with breast cancer (cases) 7912 *BRCA2* − 4091 cases	Case-Control, data from CIMBA − 33 countries including 55 centers	Weight status	Questionnaire of self-reported height and weight to calculate observed BMI at date of questionnaire and during young adulthood Included Mendelian Randomization approach: Calculated weighted genetic score for BMI and height (see paper for details)	Observed BMI and Breast Cancer Risk at Date of Questionnaire: Per 5 kg/m^2^ (participants/number of events): -All participants (6964/3331): HR, 0.94 (0.90, 0.98)* -*BRCA1* (4401/2114): HR, 0.96 (0.91, 1.01)** -*BRCA2* (3115/1480): HR, 0.90 (0.84, 0.97)** -Premenopausal (7516/2153): HR, 0.92 (0.87, 0.97)*** -Postmenopausal (3029/1389): HR, 0.97 (0.91, 1.04)*** Observed BMI and Breast Cancer Risk in Young Adulthood: Per 5 kg/m^2^ (participants/number of events): -All participants (5210/2436): HR, 0.82 (0.75, 0.90)* -*BRCA1* (3134/1462): HR, 0.87 (0.78, 0.97)** -*BRCA2* (2283/1058): HR, 0.74 (0.63, 0.85)** -Premenopausal (5417/1519): HR, 0.85 (0.78, 0.94)*** -Postmenopausal (2181/977): HR, 0.79 (0.69, 0.91)*** BMI Genetic Score and Breast Cancer Risk at Date of Questionnaire: Per 5 kg/m^2^ (participants/number of events): -All participants (22,588/11,451): HR, 0.87 (0.76, 0.98)**** -*BRCA1* (14,676/7360): HR, 0.88 (0.76, 1.02)** -*BRCA2* (7912/4091): HR, 0.83 (0.65, 1.05)** -Premenopausal (22,588/7410): HR, 0.84 (0.73, 0.98)***** -Postmenopausal (8459/3926): HR, 0.89 (0.72, 1.09)***** *Data from fully adjusted model. Adjusted for principal components, country, birth cohort, mutation status, menopausal status, parity and age at menarche. **Data adjusted for principal components, birth cohort, country of enrollment, menopausal status. *** Data adjusted for principal components, birth cohort, country of enrollment, mutation status. ****Data adjusted for principal components, birth cohort, country of enrollment, menopausal status, mutation status. *****Data adjusted for principal components, birth cohort, country of enrollment, menopausal status. Data for other multivariable adjustments and height are available in the paper.
King MC et al., 2003	104 women with *BRCA1/2* pathogenic germline gene variants 67 *BRCA1* 37 *BRCA2*	Retrospective cohort of Ashkenazi Jewish women, data from 12 participating cancer centers in the greater New York City area	Weight status Physical activity	Data collection method not provided in detail Weight status was inquired at menarche and age 21 Physical activity behavior was inquired during adolescence	Normal weight status (per BMI) at menarche (*p* = 0.017) and age 21 (*p* = 0.021) was associated with breast cancer onset at an older age*. Engagement in physical activity as a teenager was associated with breast cancer onset at an older age (*p* = 0.034)*. *Data adjusted for decade of birth of the proband.
Lammert J et al., 2018	443 matched pairs of women with *BRCA1/2* pathogenic germline gene variants	Case-control, data from 80 participating centers in 17 countries	Physical activity	Nurses’ Health Study II Physical Activity Questionnaire Standardized questionnaire including questions related to family history, medical and personal history, reproductive, hormonal and lifestyle factors	Total physical activity (moderate + vigorous) and vigorous physical activity alone was not significantly associated with breast cancer risk in adolescence (ages 12–17), young adulthood (ages 18–34), and overall (ages 12–34). Significant associations were not observed when assessed by menopausal status (i.e. pre- or postmenopausal) at breast cancer diagnosis*. Significant association was not observed for moderate physical activity among all age groups when assessed for the total sample, by postmenopausal status at breast cancer diagnosis, and among the young adulthood and overall (young adulthood + adolescence) for premenopausal status at breast cancer diagnosis. The only significant association observed was for adolescent physical activity and premenopausal at breast cancer diagnosis (see below for data)*. Moderate physical activity in adolescence and premenopausal breast cancer risk*: ≤6.75 MET-hrs/week: 1.00 > 6.75 and ≤ 15.75 MET-hrs/week: HR 1.04 (0.70,1.53) > 15.75 and ≤ 25.88 MET-hrs/week: HR 1.48 (0.94,2.32) > 25.88 MET-hrs/week: HR 0.62 (0.40,0.96) *p-trend = 0.01* *Data adjusted for number of children, current BMI, oral contraception use, tobacco consumption, and history of oophorectomy.
Pijpe A et al., 2010	558 women with *BRCA1* pathogenic germline gene variants 167 women with *BRCA2* pathogenic germline gene variants 218 carriers diagnosed with breast cancer within the 10-year period of questionnaire − 170 *BRCA1* − 48 *BRCA2*	Retrospective Cohort, HEBON study (Hereditary Breast and Ovarian Cancer Study, the Netherlands)	Physical activity	Standardized risk factor questionnaire Questions related to physical activity behavior include: type of sport, number of hours spent per week, ages at which it was practiced. Questions were specific to activities performed for at least 6 months for at least 1 h/week.	Significant associations were not observed between the following activity variables and breast cancer risk when never engaging in lifetime sports activity was the reference group: Mean MET hours/week (low, < 11.0; medium, 11.0–22.7; high, ≥22.7), Mean hours/week (low, < 2.0; medium, 2.0–3.3; high, ≥3.3), Number of active years (< 9 years, 9–19 years, ≥19 years). Lifetime sports activity and breast cancer risk *: Mean MET hours/week --low (< 11.0): 1.00 --medium (11.0–22.7): HR, 0.59 (0.36,0.95) --high (≥22.7): HR, 0.77 (0.48,1.24) *p-trend = 0.494* Significant associations were not observed for Mean hours/week and number of active years when the lowest category was used as the reference category. Lifetime sports activity before age 30 and breast cancer risk*: Mean MET hours/week --low (< 11.0): 1.00 --medium (11.0–22.7): HR, 0.60 (0.38,0.96) --high (≥22.7): HR, 0.58 (0.35,0.94) *p-trend = 0.053* Significant associations were not observed for Mean hours/week and number of active years when the lowest category was used as the reference category. Significant associations were not observed for activity variables when never engaging in lifetime sports activity was the reference group. Lifetime sports activity after age 30 and breast cancer risk*: Mean MET hours/week --never engaging in activity: 1.00 --low (< 11.0): HR, 0.55 (0.34,0.90) --medium (11.0–22.7): HR, 0.70 (0.44,1.14) --high (≥22.7): HR, 0.68 (0.43,1.09) *p-trend = 0.157* Mean hours/week --never engaging in activity: 1.00 --low (< 2.0): HR, 0.53 (0.32,0.86) --medium (2.0–3.0): HR, 0.80 (0.47,1.36) --high (≥3.0): HR, 0.66 (0.42,1.04) *p-trend = 0.135* Number of active years --never engaging in activity: 1.00 -- < 5: HR, 0.52 (0.32,0.85) --5-11: HR, 0.78 (0.48,1.26) -- ≥ 11: HR, 0.64 (0.39,1.03) *p-trend = 0.119* Sports activity --never: 1.00 --ever: HR, 0.63 (0.44,0.91) Significant associations were not observed for Mean hours/week and number of active years when the lowest category was used as the reference category. Recent sports activity and breast cancer risk by time windows: 1 year Mean hours/week --low (< 2.0): HR, 0.48 (0.26,0.87) --medium (2.0–3.0): HR, 0.90 (0.55,1.47) --high (≥3.0): HR, 0.90 (0.58,1.40) Significant associations were not observed for Mean MET hours/week or percent active years. 2 years Mean hours/week --low (< 2.0): HR, 0.49 (0.29,0.85) --medium (2.0–3.0): HR, 0.89 (0.52,1.50) --high (≥3.0): HR, 0.94 (0.61,1.44) Significant associations were not observed for Mean MET hours/week or percent active years. 5 years Mean MET hours/week --low (< 11.0): HR, 0.64 (0.42,0.98) --medium (11.0–22.7): HR, 0.91 (0.56,1.50) --high (≥22.7): HR, 0.92 (0.57,1.50) Significant associations were not observed for Mean hours/week or percent active years. 10 years Significant associations were not observed for this time window. *Data adjusted for use of oral contraceptives, parity, menopausal status, hormone replacement therapy use, age-specific BMI, BMI at age 18, alcohol consumption, occupational activity. Mean METhours/week and mean hours/week also adjusted for number of active years. Number of active years also adjusted for mean METhours/week.

*OR* odds ratio, *CI* confidence interval, *HR* hazard ratio, *COR* case-only odds ratio, *IRR* interaction risk ratio, # pounds, *MET* metabolic equivalents

Regarding weight status and weight change, after adjusting for age at menarche, parity, oral contraceptives, height, and hormone replacement therapy, McGee and colleagues [21] case-control study did not observe a significant association between current weight status or weight change throughout adulthood and diagnosis of ovarian cancer among 403 women with *BRCA1* and 66 women with *BRCA2* pathogenic germline gene variants. Risk was not assessed by *BRCA1/2* pathogenic germline gene variants individually. In contrast, Qian and colleagues [28] case-control study observed significant associations between a higher body mass index (BMI) and premenopausal ovarian cancer incidence, for both self-reported BMI (among 102 cases out of 7516 women with *BRCA1/2* pathogenic germline gene variants) and a calculated BMI genetic score (BMI-GS; among 967 cases out of 22,588 women with *BRCA1/2* pathogenic germline gene variants) based on a Mendelian Randomization approach. Higher self-reported BMI was also associated with increased risk of non-serous ovarian tumors [28]. Significant associations were not observed by *BRCA1/2* pathogenic germline gene variants, postmenopausal status, or serous tumor type [28].

### Breast Cancer risk

#### Dietary habits-alcohol consumption

Regarding women with *BRCA1* pathogenic germline gene variants In Dennis and colleagues [16] case-control study, higher alcohol consumption was associated with reduced risk (p-trend = 0.03, *n* = 1480), after adjusting for ethnicity, parity, BMI, history of oral contraceptive use, hormone replacement therapy, oophorectomy, smoking and menopausal status. Compared to non-drinkers, 0–3 drinks/week (OR 0.77, 95%CI 0.67,0.94) and ≥ 10 drinks/week (OR 0.55, 95%CI 0.33,0.91), but not 4–9 drinks/week (OR 0.98, 0.73,1.32), were significantly associated with reduced breast cancer risk [16].

In contrast, case-control studies by both Lecarpentier and colleagues [19] (*n* = 863, adjusted for parity, menopausal status, *BRCA1/2* pathogenic germline gene variant, smoking history) and McGuire and colleagues [22] (*n* = 497, adjusted for age, family history, smoking status, and full-term pregnancies), and a prospective cohort study by Cybulski and colleagues [15] (*n* = 2498 adjusted for baseline age, gene, menarche age, oral contraceptive use, breast feeding history, mean parity, oophorectomy status, and resident country) did not observe significant associations between alcohol intake and breast cancer risk among women with *BRCA1* pathogenic germline gene variant. Similarly, Moorman and colleagues [23] case-only study observed a significant, but weak effect of alcohol intake among 283 breast cancer survivors with *BRCA1* pathogenic germline gene variant compared to 891 survivors without *BRCA1* pathogenic germline gene variant (interaction risk ratio(IRR) 0.65, 95%CI 0.48,0.90) when adjusting for age and site of data collection. In Dennis and colleagues [11] case-only study (adjusted for age at diagnosis) that included 10 women with *BRCA1* pathogenic germline gene variants, significant interactions were not observed (case-only odds ratio(COR) 0.79, 95%CI 0.22,2.83).

Regarding women with *BRCA2* pathogenic germline gene variants, McGuire and colleagues [22] observed an association with ever-use of alcohol compared to never-use (OR 0.66, 95%CI 0.45,0.97), and 1–4 g/day compared to no alcohol (OR 0.41, 95%CI 0.22,0.77), among 307 women with *BRCA2* pathogenic germline gene variants. Associations were not observed for other alcohol variables (i.e. > 4 g/day, current use, years of drinking). Dennis and colleagues [11] observed a supra-multiplicative effect for all alcohol excluding wine, among 33 cancer survivors with *BRCA2* pathogenic germline gene variant compared to 814 survivors without *BRCA2* pathogenic germline gene variant (COR 2.15, 95%CI 1.03,4.49); effects were not observed for all alcohol including wine, or wine alone [11]. In contrast, other studies did not observe effects among women with *BRCA2* pathogenic germline gene variant [15, 19, 23]*.*

#### Dietary habits-coffee consumption

In Gronwald and colleagues [30] case-control study, no association was observed between coffee consumption and breast cancer risk among 348women with *BRCA1* pathogenic germline gene variant (OR 0.8, 95%CI 0.5,1.1). Amount of coffee consumption and statistical adjustments for the analysis were not specified. In contrast, Nkondjock and colleagues [24] case-control study observed an association between ≥6 cups caffeinated coffee/day among 652women with *BRCA1* pathogenic germline gene variant and breast cancer risk (OR 0.25, 95%CI 0.09,0.71). Associations were not observed for women with *BRCA2* pathogenic germline gene variant (*n* = 193). When assessing *BRCA1/2* pathogenic germline gene variants collectively, ≥6 cups/day of total coffee (caffeinated and decaffeinated; OR 0.51, 95%CI 0.26,0.98) and ≥ 6 cups/day of caffeinated coffee (OR 0.31, 95%CI 0.13,0.71) was associated with lower risk [24]. Associations were not observed for lower levels of coffee consumption or for any level of decaffeinated coffee [24]. All aforementioned results were adjusted for parity, smoking status, oral contraceptive use, alcohol and BMI at 30-years**.**

#### Dietary habits-food/nutrient intake

Ko and colleagues [12] retrospective cohort assessed dietary intake of vegetables, fruit, meat, seafood, and soybean products, in relation to breast cancer risk among 491 women with *BRCA1*/*2* pathogenic germline gene variants collectively and by pathogenic germline gene variant, and adjusted for menarche, caloric intake, years of education, smoking history, alcohol intake, exercise habits and parity. Regarding women with *BRCA1/2* pathogenic germline gene variants collectively, no association was observed with vegetable, fruit, or seafood intake. Intake of 3–10 meat food-items/day was linked with nearly doubling breast cancer risk (HR 1.97, 95%CI 1.13,3.4; p-trend = 0.026) [12]. An inverse relationship was observed for soy and breast cancer risk (HR 0.39, 95%CI 0.19,0.79, p-trend = 0.005) [12]. When assessed by *BRCA1/2* pathogenic germline gene variant, a significant positive relationship was observed for meat intake among women with *BRCA2* pathogenic germline gene variant (p-trend = 0.027), yet only the highest quartile of meat intake was associated with risk (HR 2.48, 95%CI 1.26,4.89). Moreover, an inverse relationship was observed for soy (*p* = 0.005), but only the highest quartile of soybean-product intake was associated with reduced risk (HR 0.38, 95%CI 0.16,0.93) [12]. Significant associations were not observed in women with *BRCA1* pathogenic germline gene variant.

Case-control studies conducted by Nkondjock and colleagues [31] and Kim and colleagues [27] included assessment of nutrients. Nkondjock and colleagues [31] assessed macro/micronutrient intake, alcohol, and coffee and found that among 89 women with *BRCA1/2* pathogenic germline gene variants collectively, total energy intake > 2339 kcals/day was associated with nearly tripling breast cancer risk (HR 2.76, 95%CI 1.10,7.02; p-trend = 0.026), when adjusting for age, maximum lifetime BMI, and PA [31]. Analysis was not conducted by variant. Kim and colleagues [27] assessed folic acid, B6, and B12 supplementation and observed that ever-use of a prenatal supplement was associated with reduced likelihood of breast cancer for 400 women with *BRCA1/2* pathogenic germline gene variants (OR 0.57, 95%CI 0.34,0.95), when adjusting for age and *BRCA1/2* pathogenic germline gene variant. When adjusting for age, *BRCA1/2* pathogenic germline gene variant, BMI, parity, alcohol consumption and smoking status, consumption of any folic-acid containing supplement, 8.56–89.29mcg/d of folic acid supplementation, and 0.02–0.34 mcg/d of B12 supplementation, was associated with reduced likelihood of breast cancer among women with *BRCA1/2* pathogenic germline gene variants [27]. When stratified by *BRCA1/2* pathogenic germline gene variant, significant associations were only revealed for ever-use of any folic-acid containing supplement among women with *BRCA1* pathogenic germline gene variant [27].

Nkondjock and colleagues [14] conducted a second case-control analysis assessing diet quality among 89 women with *BRCA1/2* pathogenic germline gene variants collectively. The four diet quality indexes utilized were indicative of dietary patterns and included the following: Alternative Healthy Eating Index(AHEI), Diet Quality Index-Revised(DQI-R), Alternate Mediterranean Diet Index(aMED), Canadian Healthy Eating Index(CHEI). An inverse relationship with breast cancer risk was observed for the DQI-R(p-trend = 0.034) and CHEI(p-trend = 0.006); however, only the highest tertile of CHEI was significantly associated with lower breast cancer risk (OR 0.18, 95%CI 0.05,0.68) after adjusting for age, PA and total energy intake [14].

#### Weight status/change

Studies assessing weight management evaluated adulthood and young-adulthood weight status, and adulthood weight change. Moorman and colleagues [23] did not observe effects between BMI one year before diagnosis or at age 18 and breast cancer risk. Among retrospective cohorts, Manders and colleagues [20] observed, among 218 women with *BRCA1/2*pathogenic germline gene variants, that risk doubled when current weight was ≥72 kg (HR 2.10, 95%CI 1.23,3.59). These findings were observed in a time-varying Cox-proportional hazard model stratified by gene and birth cohort, clustered for family, and adjusted for parity, menopausal status, hormone replacement therapy, and lifetime sports activity [20]. Among 104 Ashkenazi Jewish women, normal BMI at menarche and 21-years significantly delayed age of onset of breast cancer after adjusting for pro-band decade of birth [17]. Nkondjock and colleagues [31] observed that women with *BRCA1/2* pathogenic germline gene variants who experienced their maximum BMI at > 43-years were at a nearly 3-fold increased risk of breast cancer (OR 2.90, 95%CI 1.01,8.36).

Among case-control studies, Abbas and colleagues [26] observed a significant association with adulthood overweight/obesity status and increased likelihood of breast cancer (overweight: OR 3.06, 95%CI 1.36,6.87; obesity: OR 4.09, 95%CI 1.91,8.75) among 200 women with *BRCA1/2* pathogenic germline gene variants. In contrast, Qian and colleagues [29] observed a reduction in breast cancer risk with each 5 kg/m^2^ increase in BMI at adulthood, young-adulthood, and BMI-GS analyses among women with *BRCA1/2* pathogenic germline gene variants (adulthood: HR 0.94, 95%CI 0.90,0.98; *n* = 6964; 3331 events; young-adulthood: HR 0.82, 95%CI 0.75,0.90; *n* = 5210; 2436 events; BMI-GS: HR 0.87, 95%CI 0.76,0.98; *n* = 22,588; 11,451 events) [29]. For self-reported BMI, when stratified by *BRCA1/2* pathogenic germline gene variant, adulthood BMI was inversely associated with breast cancer risk for women with *BRCA2* pathogenic germline gene variant, but not *BRCA1* pathogenic germline gene variant, and young-adulthood BMI was inversely associated with risk among women with *BRCA1* and *BRCA2* pathogenic germline gene variants [29]. Similar results were observed when stratified by menopausal status, such that significant inverse associations were observed for adulthood BMI, BMI-GS and premenopausal breast cancer risk, and young adulthood BMI and both pre- and postmenopausal breast cancer risk [29].

Regarding adulthood weight gain, Nkondjock and colleagues [31] observed a positive relationship for weight gain since age 18 (p-trend = 0.011) and 30 (p-trend = 0.013) and breast cancer risk. Women with *BRCA1/2* pathogenic germline gene variants who gained 12–35 pounds since age 18 exhibited 3.6-fold increased risk (OR 3.63, 95%CI 1.18,11.22) and women who gained > 35 pounds exhibited 4.6-fold increased risk (OR 4.64, 95%CI 1.52,14.12) [31]. Since age 30, women who gained 9–20 pounds presented 3.4-fold increased risk (OR 3.43, 95%CI 1.16,10.14), and women who gained > 20 pounds displayed 4-fold increased risk (OR 4.11, 95%CI 1.46,11.56) [31]. In contrast, Kotsopoulos and colleagues [18] case-control study did not observe a significant relationship for weight gain between ages 18–30 among 1073 women with *BRCA1/2* pathogenic germline gene variants [31]. Additionally, Manders and colleagues [20] did not observe a relationship between adult weight change and pre- or post-menopausal breast cancer risk.

Interestingly, Kotsopoulos and colleagues [18] considered adulthood weight loss between ages 18–30. A significant association was observed between loss ≥10 pounds and decreased breast cancer risk (OR 0.66, 95%CI 0.46,0.93) [18]. When assessed by *BRCA1/2* pathogenic germline gene variant, a significant association was observed between weight loss of ≥10 pounds and reduced risk of breast cancer among women with *BRCA1* but not *BRCA2* pathogenic germline gene variant [18].

#### Physical activity

Studies assessing PA, evaluated activity across varying time periods, among women with *BRCA1/2* pathogenic germline gene variants collectively. Among Ashkenazi Jewish women, engagement in PA as a teenager was associated with delayed onset of breast cancer [17]. Nkondjock and colleagues [31] did not observe significant associations between PA variables two years before breast cancer diagnosis and breast cancer risk.

Alternatively, Lammert and colleagues [13] and Pijpe and colleagues [25] evaluated PA over longer periods of time. Lammert and colleagues [13] case-control study among 433 women with *BRCA1/2* pathogenic germline gene variants assessed PA in adolescence and early adulthood and adjusted analyses for number of children, current BMI, history of oral contraceptive use and/or oophorectomy, and tobacco consumption. Pijpe and colleagues [25] retrospective cohort among 725 women with *BRCA1/2* pathogenic germline gene variants assessed lifetime sports activity and adjusted for oral contraceptives, parity, menopausal status, hormone replacement therapy, age-specific BMI, BMI at age 18, alcohol consumption, occupational activity. In certain analyses, mean metabolic equivalent (MET)-hours/week and mean hours/week were adjusted for number of active years, and number of active years were also adjusted for mean MET-hours/week.

Lammert and colleagues [13] assessed moderate, vigorous, and total activity in MET-hours/week in adolescence, young adulthood, and overall. Analysis was also stratified by menopausal status at diagnosis. The only association observed was for the highest quartile of moderate activity in adolescence, > 25.88 MET-hours/week, in relation to premenopausal breast cancer risk (HR 0.62, 95%CI 0.40,0.96) [13].

Pijpe and colleagues [25] assessed lifetime sports activity overall, before and after age 30, and in time windows (one-year, two-, five-, and 10-years) before age 35. Overall, 11–22.7 MET-hours/week of sports activity was associated with a 41% reduction in risk (HR 0.59, 95%CI 0.36,0.95), whereas ≥22.7 MET-hours/week was not associated with reduced risk (HR 0.77, 95%CI 0.8,1.24) [25]. When never engaging in sports activity was the reference, significant associations were not observed [25].

Before age 30, when the lowest sports activity category was used as the reference, 11–22.7 and ≥ 22.7 mean MET-hours/week was associated with a 40% reduction in breast cancer risk [9, 25]. Associations were not observed when never-engaging in sports activity was used as the reference category. In contrast, after age 30, ever-engaging in sports activity was associated with a 37% reduction in breast cancer risk (HR 0.63, 95%CI 0.44,0.91 [25]. When never-engaging in sports activity was the reference category, significant associations were only observed for the lowest category (least amount of activity) of each variable [32].

Table 3 provides a summary of results for diet, weight, and PA in relation to ovarian and breast cancer risk among women with *BRCA1/2* pathogenic germline gene variants.

**Table 3:** Diet/Weight/Physical Activity and Ovarian & Breast Cancer Risk in *BRCA1/2* Pathogenic Germline Gene Variant Carriers

Cancer Type	Energy Balance-Related Factors	Major Findings
Ovarian Cancer	Dietary Habits	• 1 study; No association between regular coffee consumption and ovarian cancer risk in *BRCA1*^(Gronwald, 2006)^
	Weight Status/Weight Change	• 2 studies ^(McGee, 2012; Qian, 2019)^ • No association between weight change in adulthood and ovarian cancer risk in *BRCA1* & *BRCA2*^(McGee, 2012)^ • Significant association between higher BMI and premenopausal ovarian cancer risk *BRCA1* & *BRCA2*^(Qian, 2019)^
Breast Cancer	Dietary Habits	• 12 studies^a^ • Decreased Breast Cancer Risk: o Significantly associated with higher intakes of caffeinated coffee in *BRCA1/2 & BRCA2*^b,c (Nkondjock, Ghadirian, 2006)^ o Significantly associated with higher intake of soybean foods in *BRCA1/2 & BRCA2*^b,c (Ko, 2013)^ o Significantly associated with higher diet quality in *BRCA1/2*^b (Nkondjock A et al, 2007)^ o Significantly associated with folic acid and B12 supplementation at specific doses in *BRCA1/2*^d (Kim, 2019)^ o Significantly associated with any folic acid containing supplement in *BRCA1*^e (Kim, 2019)^ • Increased Breast Cancer Risk: o Significantly associated with higher intake of meat in *BRCA1/2 & BRCA2*^b,c (Ko, 2013)^ o Significantly associated with higher daily energy intake (> 2339 kcal/d) in *BRCA1/2*^(Nkondjock, Robidoux, 2006)^ • Evidence related to total coffee consumption (caffeinated and decaffeinated) is mixed^f^ • Evidence related to alcohol intake is mixed^g^ • No association between macro/micro-nutrient intake and breast cancer risk in *BRCA1/2*^b(Nkondjock, Robidoux, 2006)^
	Weight Status/Weight Change	• 7 studies^h^ • Decreased Breast Cancer Risk: o Significantly associated with ≥10-lb weight loss between 18 & 30 years in *BRCA1/2*^b (Kotsopoulos, 2005)^ o Significantly associated with higher BMI in young adulthood in *BRCA1/2, BRCA1, BRCA2*^*b,c,e,* i, (Qian, 2019)^ • Increased Breast Cancer Risk: o Significantly associated with adulthood body weight ≥ 72 kg & postmenopausal breast cancer risk in *BRCA1/2*^b (Manders, 2011)^ • Evidence related to adulthood weight gain and breast cancer risk is mixed^j^ • Evidence related to overweight/obesity status in adulthood and breast cancer risk is mixed^k^ • No effect observed for BMI at 18 and BMI one year before diagnosis and breast cancer risk^b (Moorman, 2010)^ • For Ashkenazi Jewish women, normal weight status at menarche and age 21 associated with delayed onset of breast cancer^(King, 2003)^
	Physical Activity	• 4 studies • Decreased Breast Cancer Risk: o Significantly associated with activity during adolescence, high levels of activity before age 30, and lower levels of activity after age 30 in *BRCA1/2*^b(Lammert, 2018; Pijpe, 2010)^ • No association for activity two years before diagnosis and breast cancer risk in *BRCA1/2*^b(Nkondjock,Robidoux, 2006)^ • For Ashkenazi Jewish women, engagement in physical activity as teenager associated with delayed onset breast cancer^(King, 2003)^

*lb* pound; *BMI* body mass index

^a^Seven studies assessed alcohol intake (6 exclusive to alcohol, 1 included alcohol with nutrient intake), two assessed coffee intake, one assessed supplement use (folic acid, B6, B12), one assessed food group intake, one assessed nutrient intake (and included alcohol), one assessed diet quality

^b^Both *BRCA1* and *BRCA2* pathogenic germline gene variants combined in the analysis

^c^Only *BRCA2* pathogenic germline gene variant in the analysis

^d^Folic acid:8.56- ≤ 89.29mcg/d; B12:0.02- ≤ 0.34mcg/d

^e^Only *BRCA1pathogenic germline gene* variant in the analysis

^f^One study observed no association(Gronwald, 2006) and one study observed OR0.51(0.26,0.98) for total coffee consumption in relation to breast cancer risk(Nkondjock, Ghadirian, 2006)

^g^Three studies observed no association between alcohol intake and breast cancer risk in *BRCA1/2* variants collectively(Cybulski, 2015; Nkondjock, Robidoux, 2006; Lecarpentier 2011), one study observed an association in *BRCA1* but not *BRCA2* when tobacco use was included as an interaction(Lecarpentier, 2011), one study observed an association in *BRCA1* but not *BRCA2*(Dennis, 2010), one study observed a weak effect of alcohol when comparing breast cancer survivors compared to survivors without *BRCA,* no effect was observed for *BRCA2*(Moorman, 2010), one study observed an association in *BRCA2* but not *BRCA1(*McGuire, 2006), one study observed an effect for alcohol when comparing survivors with *BRCA2* to survivors without *BRCA*, but an effect was not observed in *BRCA1*(Dennis, 2011)

^h^One study(King, 2003) assessed weight status and physical activity

^i^Association applies to pre- and post-menopausal breast cancer risk

^j^One study observed a significant association with weight gain since age 18 and 30 and increased breast cancer risk for *BRCA1/2* variants (Nkondjock, Robidoux 2006), one study did not observe a significant association with 10–20 or > 20 lb. weight gain between the ages of 18 and 30 for *BRCA1/2* variants collectively and by variant, and when age at diagnosis was between 30 and 40 years or > 40 years (Kotsopoulos, 2005)

^k^One study observed a significant inverse association between breast cancer risk and self-reported adulthood overweight/obesity and genetically scored overweight/obesity (Qian, 2019), one study observed a significant positive association between breast cancer risk and adulthood overweight/obesity(Abba, 2019), one study observed a significant positive association between breast cancer risk and adulthood overweight/obesity beyond age 43(Nkondjock, Robidoux, 2006), one study observed a significant positive association with postmenopausal breast cancer risk and adulthood body weight ≥ 72 kg(Manders, 2011)

## Discussion

This systematic review did not find cohesive evidence supporting the need for tailored recommendations regarding dietary habits, weight management and PA for ovarian and breast cancer risk-reduction among women with *BRCA1* or *BRCA2* pathogenic germline gene variants*.* Regarding ovarian cancer risk, there was limited evidence supporting relationships between dietary habits and ovarian cancer incidence. The limited findings related to weight management and premenopausal ovarian cancer risk are similar to findings observed in the general population, which suggests probable relationship between body fatness and increased risk of ovarian cancer [33].

Among the general population, evidence is probable and convincing that alcohol intake increases pre- and postmenopausal breast cancer risk, respectively [34]. Thus current cancer prevention guidelines recommend limiting alcohol [34]. Among women with *BRCA1/2* pathogenic germline gene variants collectively and by *BRCA1/2* pathogenic variant, evidence is mixed [11, 15, 16, 19, 22, 23]. Notably, some studies demonstrated no association between alcohol intake and breast cancer risk among women with *BRCA1/2* pathogenic germline gene variants collectively [15, 19, 31], while others observed a reduction in risk with alcohol intake in women with *BRCA1* pathogenic germline gene variants [16, 23] and *BRCA2* pathogenic germline gene variants [22]. Considering the known potential harms associated with alcohol, stronger and more consistent evidence is needed to support more liberal guidelines for alcohol use in women with *BRCA1/2* pathogenic germline gene variants.

Evidence is limited related to food/nutrient intake and breast cancer risk among women with *BRCA1/2* pathogenic germline gene variants [12, 14, 27, 31]. An association was not observed with vegetable intake [12], and findings for micronutrients are mixed, pending the nutrient [27, 31]. Among the general population, evidence is limited and inconclusive regarding the relationship between non-starchy vegetables, nutrients, and breast cancer risk [34].

Evidence is also mixed for adulthood weight gain [18, 20] and adulthood weight status [20, 26, 29, 31]. Among the general population, evidence is probable that overweight/obesity in young adulthood decreases pre- and postmenopausal breast cancer risk, and overweight/obesity in adulthood increases postmenopausal breast cancer risk [34]. Whether weight management recommendations should differ for women with *BRCA1/2* pathogenic germline gene variants remains elusive.

Regarding PA, activity in adolescence and lifetime activity appear to have some association with breast cancer risk-reduction among women with *BRCA1/2* pathogenic germline gene variants collectively [13, 25]. This notion is supported by evidence from the general population, such that it is probable that PA, regardless of intensity, reduces postmenopausal risk and vigorous-intensity activity reduces premenopausal risk [34]. Thus, activity recommendations should remain consistent with recommendations for the general population.

This is the first study to our knowledge to systematically evaluate whether tailored recommendations related to dietary habits, weight management and PA may be effective in reducing ovarian and breast cancer risk among women with *BRCA1/2* pathogenic germline gene variants. We consider the following factors limitations of the current state of evidence: small number of studies for both ovarian and breast cancer risk in this high risk population, especially when considering the available large epidemiological studies that have established associations of lifestyle factors among the general population; heterogeneity in methods to evaluate lifestyle factors; inconsistent confounding factors; no data evaluating hormone receptor status; limited data evaluating by gene variant and menopausal status. Considering these limitations, notably the heterogeneity of the current evidence, inability to separate analyses by *BRCA1/2* pathogenic germline gene variant, and retrospective nature of the majority of studies conducted, it is difficult to determine the extent of which recommendations for lifestyle factors should differ for this higher risk population. Future observational studies should address these limitations, specifically prospective, larger cohort studies enabling one to assess risk for these factors by gene variant.

## Conclusions

Among women with *BRCA1/2* pathogenic germline gene variants, there is insufficient evidence for recommendations related to dietary habits or weight management and ovarian cancer risk. Pertaining to breast cancer, there is not enough evidence to suggest variation from current recommendations for the general population for dietary habits or weight management. There is no evidence to suggest that risk association related to physical activity differed from the general population; therefore, recommendations for physical activity should remain the same.

## Supplementary information

**Additional file 1.** Search Strategy.

## Data Availability

Not applicable.

## Abbreviations

AHEI: Alternative Healthy Eating Index
aMED: Alternate Mediterranean Diet Index
BMI: Body Mass Indez
BMI-GS: Calculated Body Mass Index- Genetic Score
CHEI: Canadian Healthy Eating Index (CHEI)
COR: Case-Only Odds Ratio
DQI-R: Diet Quality Index-Revised
IRR: Interaction Risk Ratio
MET: Metabolic Equivalent
PA: Physical Activity
PRISMA: Preferred Reporting Items for Systematic Reviews and Meta-Analyses
