# Supplementary material for: Diet, weight management, physical activity and Ovarian & Breast Cancer Risk in women with BRCA1/2 pathogenic Germline gene variants: systematic review
Source: Hered Cancer Clin Pract. 2020 Mar 7;18:5. doi: 10.1186/s13053-020-0137-1 (PMC7060535; doi:10.1186/s13053-020-0137-1)
Supplement: Supplementary file 1 — Additional file 1. Search Strategy. [file 13053_2020_137_MOESM1_ESM.docx]

**Ovid MEDLINE(R) ALL (Ovid MEDLINE and In-Process & Other Non-Indexed Citations, 1946 to Present). Searched on October 3, 2019.**

1. BRCA1 Protein/
2. BRCA2 Protein/
3. Genes, BRCA1/
4. Genes, BRCA2/
5. (BRCA* or "breast cancer type 1" or "breast cancer type 2" or "breast Cancer 1" or "breast cancer 2").ti,ab,kf.
6. or/1-5
7. exp Breast Neoplasms/
8. (breast adj4 (adenoma* or cancer* or carcinoma* or metasta* or neoplas* or sarcoma* or tumo?r)).ti,ab,kf.
9. exp Ovarian Neoplasms/
10. ((ovarian or ovary or ovaries) adj4 (cancer* or carcinoma* or metasta* or neoplasm* or sarcoma* or tumor* or tumour*)).ti,ab,kf.
11. or/7-10
12. 6 AND 11
13. exp Energy Metabolism/
14. ("energy balance" or "energy imbalance" or "energy expenditure" or "energy intake" or metabolic or metabolism)
15. exp Exercise/
16. exp Exercise Therapy/
17. exp Physical Endurance/
18. Physical Exertion/
19. exp Physical Fitness/
20. Sedentary Lifestyle/
21. exp Sports/
22. Walking/
23. (aerobic* or endurance or exercis* or fitness* or "physical activit*" or "physical exertion" or sedentary or inactiv* or walking).ti,ab,kf.
24. Health Behavior/
25. Healthy Lifestyle/
26. exp Life Style/
27. ("health behavior*" or "health risk behavior*" or lifestyle* or "life style*").ti,ab,kf.
28. exp "Diet, food, and nutrition"/
29. exp "Energy Intake"/
30. Feeding Behavior/
31. Food Preferences/
32. exp "Healthy Diet"/
33. (caloric or coffee or caffein* or diet or dietary or eating or "energy consumption" or "energy intake" or feeding or food* or fruit* or meat or nutrient* or nutrition* or overfeed or "over feed" or over-nutrition or soy or soybean* or vegetable*).ti,ab,kf.
34. exp "Dietary supplements"/
35. "Minerals"[nm]
36. Exp Minerals/
37. exp Vitamins/
38. ("multi-mineral*" or multimineral* or multivitamin* or vitamin* or supplement*).ti,ab,kf.
39. Exp Alcohol Drinking/
40. Alcoholism/
41. ("alcohol abuse" or "alcohol consumption" or "alcohol dependence" or "alcohol intake" or "alcohol use" or alcoholic* or alcoholism or "daily alcohol" or dr?nk or drinking).ti,ab,kf.
42. Adiposity/
43. exp Body fat distribution/
44. exp Body Mass Index/
45. Body Size/
46. exp Body Weight/
47. (Adipos* or BMI or "body composition" or "body fat" or "body mass" or "body size" or "body weight" or "Fat deposition" or obes* or "over-weight" or overweight or underweight or "waist-hip ratio" or weight).ti,ab,kf.
48. OR/13-47
49. exp Risk/
50. exp Causality/
51. exp Epidemiologic Studies/
52. (association or "before after" or causa* or "case control" or cause* or cohort* or comparative or correlation* or correspond* or "cross sectional" or determinant* or epidemiol* or factor? Or "follow-up" or link? or longitudinal or meta-analysis or predict* or prospective or retrospective or risk? or "systematic review").ti,ab,kf.
53. OR/49-52
54. 12 AND 48 AND 53
55. exp animals/ not exp humans/
56. (mouse or mice or rat or rats).ti,ab,kw.
57. or/55-56
58. 54 not 57
59. limit 58 to english language
